# (Re‐) Defining evolutionary medicine

**DOI:** 10.1002/ece3.6825

**Published:** 2020-10-01

**Authors:** Jacqueline Moltzau Anderson, Florian Horn

**Affiliations:** ^1^ Department of Microbiology and Immunology University of Michigan Medical School Ann Arbor MI USA; ^2^ Kiel Germany

**Keywords:** development and evolution, education, evolutionary medicine, evolutionary theory, molecular evolution, phylogeography, pre‐medical curriculum

## Abstract

The applicability of evolutionary biology principles to diseases has been largely questioned by the medical field. While Evolutionary Medicine (EM) developed in part to lessen this gap, EM is an independent field from both evolution and medicine, whose continued narrowing of topics as a consequence of its reductionist approach, in addition to its focus to introduce itself at a late stage in medical education, has led to its continued resistance toward implementation. In turn, this has had a profound and lasting impact on the awareness of evolution in medicine among physicians. For both the evolutionary and medical communities to reach a common perspective and obtain a greater frame‐work of medical thought, a comprehensive view of the evolution of the healthy human being needs to be introduced as a starting point during the premedical curriculum. Here, we present our views on the ongoing challenges that have caused the continued division between the evolutionary fields and medicine, and provide solutions to help bridge the gap for an interdisciplinary field of evolution in medicine.

## Graphical abstract

The applicability of evolutionary biology principles to diseases has been largely questioned by the medical field. Although support has grown to introduce Evolutionary Medicine (EM) into the medical curriculum, the profoundly different perspectives and approaches of these fields, as well as its late introduction in medical education, continue to hinder this process. This makes evolution (rather than EM) easier to integrate into medicine and can be achieved when the application of a comprehensive view of the evolution of the healthy human being is implemented in the pre‐medical curriculum, and the system of rewards is developed.

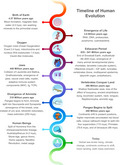

## 1 EVOLUTION AND MEDICINE

In the early 1900s, the practice of medicine underwent a transformation through its integration with the natural sciences, which developed in parallel, but largely independently and could be directly applied to explain disease. With the addition of the basic sciences, scientific explanations of the etiology and pathogenesis of diseases, increasingly found their way into medical practice, where previously only practical experiences could be drawn upon. Today, these basic sciences have become an established and required part of the medical student's curriculum (Varki, 2012), whether in the form of a premedical degree, known as “pre‐med” in the United States, or by direct preclinical training, as required in Germany and Austria, as part of medical studies. In both the premedical studies and preclinical training, hereafter referred to as the premedical curriculum, the core subjects (such as anatomy, physiology, and biochemistry) are taught prior to the practical training “at the bedside.” Supplemented with a clinical‐theoretical part (pharmacology, pathology, and microbiology), which also precedes the clinical‐practical training, modern medicine is an empirical science, which is thoroughly based on the natural sciences. Why then is evolutionary biology missing? While both Jean‐Baptiste Lamarck's (Lamarck, 1801) and Charles Darwin's (Darwin, 1859) theories had preceded medicine's transformative stage, evolutionary biology remained unrecognized as a field of study and was never integrated into the medical curriculum (Varki, 2012).

Today, while there are some traces of evolutionary concepts in the courses for medical students, there is no evolution in medicine, nor is there evolutionary medicine (EM) in medicine. Take for instance, antibiotic resistance. While commonly acknowledged and an urgent global concern, the principles for how antibiotic resistance emerges, can be transmitted, or the rate of emerging resistance within an individual is largely unknown by physicians. By applying standard medical protocols (identifying the infectious organism and administering the appropriate therapy), physicians reach the same end result, whether or not the evolutionary principles are understood. Despite the progress that has been made through strong advocacy for educational reform in medicine, there continues to remain a lack of interest and action to include evolutionary explanations in medicine.

Here, we present key problems with integrating the evolutionary fields (evolution and EM) into medicine, explain why this process is failing and provide some possible solutions. We will particularly focus on the difference in perspective between the fields, emphasized by “how” and “why” questions, and promote the understanding that a middle ground exists where both evolution and medicine can meet through the concept of a comprehensive view of the evolving healthy human being.

## 2 EVOLUTIONARY MEDICINE

EM emerged as a field to aid the progress of medicine by applying evolutionary principles to understand health and disease. One of the most valuable insights EM has reintroduced to medicine is the principle of evolutionary compromise, whereby adaptations tend to be constrained by tradeoffs. Take the famously used example of the human eye, which has numerous constraints as its novelties, or adaptations, and is built from a foundation with limited possibilities. The current constraints of the human eye are the result of adaptations over time for which only certain outcomes were possible due to its original construct. Yet, herein lies a fundamental difference between the evolutionary fields (EM and evolution) and medicine. These compromises have taken place through selection over long periods of time, and while EM and evolution rely on these time scales, medicine does not.

## 3 “WHY” AND “HOW” QUESTIONS

Compared to physicians, the EM and evolutionary biology communities have a fundamentally different perspective of time. Medicine is restricted to a level of causation that is extremely short, otherwise known as proximate causes, or “how” questions (Alcock & Schwartz, 2011). Consider a linear timescale; medicine most often deals with the present symptoms (acute symptoms), it may then consider the patient's history (individual's lifetime and chronic symptoms), and at its farthest point, the patient's parents (hereditary diseases and genetic predispositions causing higher disease risk). On the other hand, evolutionary explanations seek to understand “why” traits have evolved. In the practical sense, imagine the following scenario. A patient diagnosed with cancer may ask their physician, “why did I get cancer?”. A physician will often answer, “because you have a specific mutation(s).” However, this does not answer the patient's question. They are not asking “how I got cancer?”, referring to the proximate cause, but “why” referring to the ultimate cause, or evolutionary explanation. Why did the cancer arise to begin with? Contrary to a physician, an EM member might say “it is a shared consequence of multicellular life.” In such cases, the proverb “timing is everything” could not be more relevant to distinguish this difference in perspective.

## 4 PROBLEMS IN EVOLUTIONARY MEDICINE

Although EM and evolution share the core principles of evolutionary biology, they remain distinct from one another. Perhaps as a result of EM's primary focus in medicine, EM has developed an emphasis on a few topics, while the rest are aggregated into different, but separate fields. On the other hand, evolution is integrated into these other fields (such as veterinary medicine, paleopathology, evolutionary developmental biology, and evolutionary or biological anthropology) and EM has become another branch. This increases the difficulty of bringing these disciplines together into medicine.

Moreover, EM's emerging trend of concentrated topics dealing with medicine is subsequently also the ones that are not working with the evolution of humans. Ultimately, human evolution occurs too slowly and is often the root of the shared problem for why it is so challenging to apply evolution, or EM as it is currently being taught, into the medical curriculum. Undoubtedly, it is easier to study the evolution of pathogens, which can produce new generations in as little as 20 minutes. Whereas it is much more challenging to explain to a physician and the medical community why they should care about the evolution of humans when neither they, nor their patient, will be around to see or experience these changes.

Another fundamental difference is the approach between the evolutionary fields and medicine. Evolution and EM both apply a methodological approach where the principles of evolutionary biology are central to its process. In this way, evolutionary content is applicable to any subject or organism and why it can successfully branch off, or be implemented, into other fields. On the other hand, medicine uses a reductionist approach relying on content, where learning to identify diseases through patterns of signs of symptoms comes first. This in turn limits medicine to humans and specific diseases, leaving no room for additional material that does not have any direct relevance for the clinic (Why should a physician learn about frogs? That is what a veterinarian is for!). Thus, while EM applies evolutionary biology, its focus on health and disease has caused it to take medicine's reductionist approach. Understanding this methodological difference is critical for the future success of implementing evolution in medicine.

Finally, although efforts have been made to integrate EM into the medical curriculum, as it currently stands, EM is taught almost exclusively to evolutionary biologists. Few physicians have been exposed to evolution during their premedical or medical studies and fewer still have had the opportunity to apply it to their work. If EM continues in its current fashion, it will become exclusively research centered, for which only the results, or proximate causes, will be of interest to physicians.

## 5 THE HEALTHY HUMAN BEING

To tackle these differences and integrate evolution and medicine, a comprehensive view of the evolution of the healthy human being needs to be introduced in the premedical curriculum and would serve as a middle ground to build upon a common perspective. EM's concrete starting point is the question of a disease; “Why we get sick?”. While this may emulate medicine, EM is not based on a previous understanding of the healthy human being equivalent to the medical field of anatomy. On the other hand, medicine includes the healthy human being as a prerequisite for understanding disease, yet evolution is completely absent. The commonality would be to address the question of why a design characteristic causing susceptibility to a diseases arose, after developing an understanding of the evolution of the healthy human being. Through this application, light would be shed on the evolutionary role and function of normal processes, and on limitations and disease. Just as the core subjects are essential to the premedical curriculum, so too should evolution be essential to medicine.

## 6 ADDING THE HEALTHY HUMAN BEING

Taking a closer look at the core premedical subjects demonstrates that evolution could be easily integrated, with only a few topics requiring a new approach for teaching the material. For instance, biology is fundamental to teaching the basic evolutionary background and phylogeny systematically. Anatomy should begin with the evolution of the human organ system integrated with human embryology and closely tied to evolutionary developmental biology. Histology, another component of anatomy would need to expand its understanding of the evolution of cells, which is currently lacking and has not been adequately addressed. Biochemistry, otherwise understood as molecular medicine, would further transition to evolutionary molecular medicine (Nesse et al., 2012), and currently has the most direct influence on medicine (e.g., porphyria and porphyrins). The benefits for the systematic integration of evolution in medicine would be the exposure and a broadening of the understanding of the human being through its evolutionary history. In turn, this would provide new insights through different approaches to answer longstanding questions of disease.

To obtain an in‐dept understanding, the evolutionary history is needed and should be implemented to the premedical curriculum. With evolution clearly underpinning the core subjects, anatomy and biochemistry are great starting points. The integration of the fields would also be most cohesive to introduce prospective physicians to evolution and the notion that humans have an evolutionary history. This would also provide premedical students with time to understand the clinical relevance, ask “why” questions, and in the future to apply this knowledge in the clinic, toward research, and all areas of specialties. The premedical curriculum is so important for implementing evolution, that it becomes nearly impossible to introduce the material later with the intent for physicians to apply it. However, to apply the evolution of the healthy human being, phylogenies are needed as a prerequisite.

## 7 THE PHYLOGENY OF “MAN” IS MORE THAN THE “HUMAN HISTORY”

**FIGURE 1:**
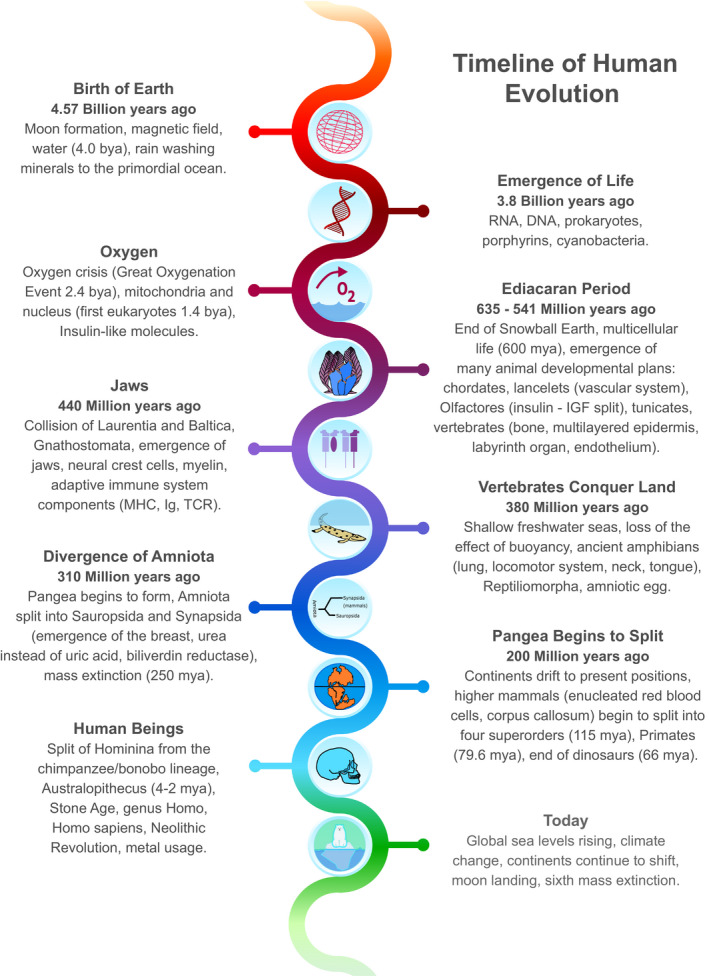
Timeline of human evolution. Inclusive timeline beginning with the birth of Earth and continuing to present day events. Events selected include environmental, biological, chemical, molecular, and anatomical developments from a wide range of evolutionary fields that have had a direct impact on humans and subsequently, an important consequence on modern medicine

Phylogenies serve an important purpose of demonstrating the missing time scale in medicine. However, while phylogenies are completely missing from medicine, they are too restricted in EM, in both time and events, and should begin from the birth of Earth itself in order to incorporate important processes that have impacted all life on Earth (Figure 1). Despite these problems, phylogenies do provide an example of why evolution should be implemented, rather than EM, and could serve as an initial solution to bridging the gap between evolutionary biologists and physicians.

Usually, discussing the “evolution of man” refers either to the beginning of the divergence of chimpanzees and man, to the *Homo* lineage, or exclusively to *Homo sapiens*. However, most of the relevant events take place prior to the emergence of our species. Rather, they begin with the development of life itself. As such, current phylogenies are often incomplete and cannot address medically relevant questions that would require a much greater historical timescale. Additionally, phylogenies tend to focus solely on biology, excluding fundamental geological and chemical processes that have also contributed to life as we know it. Yet, the birth of Earth took place first, with the environment preceding the development of life. For instance, the early oceans were essential for the initial development of life, the eventual emergence of Cyanobacteria, which in turn led to the rise of oxygen, the use of Iodine, the development of Glutathione, and multicellularity, which have many clinically relevant implications today (Figure 1). The emergence of insulin‐like substances has been shown to have occurred as early as unicellular organisms (Le Roith et al., 1980; Vitali et al., 2018), and the clinically relevant common history of insulin and insulin‐like growth factor diverged prior to when vertebrates arose (McRory & Sherwood, 1997). The addition of phylogeographical events (plate tectonics) further improves the understanding of changes that have had a role in medicine today, such as the kidney's response to freshwater and saltwater changes, and geographical disease risk in population groups (such as malaria, Crohn's disease, and hemochromatosis).

For these reasons, it is essential to expand the focus of current phylogenies to begin with the birth of Earth and complement it with geographical and chemical events. However, phylogenies need to be taught through the medical lens and should only focus on events, or content, that directly impact humans (Figure 1). Although this does narrow the countless possibilities offered by a phylogeny, it would help to reach a compromise between evolution and medicine by keeping the scope of the content relevant to physicians and patients.

## 8 BENEFITS OF IMPLEMENTING A COMPREHENSIVE VIEW OF THE EVOLUTION OF THE HEALTHY HUMAN BEING

A clear benefit of implementing phylogenies with the concept of the evolution of the healthy human being is the deeper understanding of the human body, which reduces the complexity of learning material through shared similarities (Nesse et al., 2010; Nesse & Dawkins, 2010). If one common origin leads to the development of similar structures, it is easier to both teach and learn the common origin than it is to know all of its subsequent individual features. Consider Oxytocin and Adiuretin, which have overlapping properties (similar side effects when used in the clinic) resulting from a common shared gene and a subsequent gene duplication. Thrombocytes and Erythrocytes also serve as a good example, where a common evolutionary history gave rise to overlapping properties. In terms of the clinical benefits, understanding the shared common origins also applies, such as the similar side‐effects of the steroid hormones cortisol and aldosterone. Regarding research, the benefit lies not only in a better understanding, but also in a new perspective altogether. It expands upon already existing research on proximate causes, by providing new insights into the reasons behind such causes through ultimate explanations. This delivers new answers as to why we have more antibiotic‐resistant pathogens, but also why this trend developed and how to find viable, long‐term solutions. However, this benefit is not restricted to microbial pathology and would incorporate a wider network of physicians in medically centered research areas that would directly benefit from the addition of evolution (such as research on the development of organs and systems).

## 9 EM'S DISCOURSE CHALLENGES

A common challenge that arises in any field is the challenge of interdisciplinary discourse. This is particularly true when implementing new subjects into an already existing curriculum. Although EM and physicians may share the same aim, it does not mean that they share the same perspective on how to approach it. Moreover, as new methods and knowledge develop and accumulate, the subject becomes defined as a separate field altogether. Unsurprisingly, most research, and consequently academic publications, are in a different field from medicine. However, if evolution is going to integrate into medicine, then an increased exchange between evolutionary biologists and physicians is needed, regardless of the inter‐ and transdisciplinary problems that will occur (Dahm et al., 2019). To resolve this, a system of rewards, or credit, to motivate interdisciplinary discourse between evolution and medicine is essential.

## 10 SYSTEM OF REWARDS

To address the first challenge, steps need to be implemented to begin to introduce evolutionary research into medical journals. This is not limited to just new works being published, but also to prior works that are currently unrecognized by physicians. Hypotheses on the evolution of every organ system have already been published, yet they remain unrecognized to the medical field. There is also value to reviewing older publications that are relevant for today. Consider Mendel's laws being rediscovered, or that of Aristotle, or Hildegard von Bingen. Unfortunately, much of this work is not considered research, and consequently is not given any funding or importance.

Without a system of rewards in place, there will be no motivation to act toward adding evolution to the premedical curriculum. In a survey of North American medical schools in 2003, only four hours were implemented into the curriculum for core topics of evolution (20% of deans reported 0 hr), although 48% of medical school deans stated that understanding evolutionary concepts is important for physicians (Nesse & Schiffman, 2003). While both the Association of the American Medical Colleges and the Howard Hughes Medical Institute recommended evolutionary thinking as a core competency for premedical education, it remains absent from most medical school curriculums (AAMC‐HHMI Committee, 2009; Grunspan et al., 2018). In a 2013 follow‐up study, curriculum deans for all North American medical schools were invited to participate in a survey. Notably, only 60 schools took part of which the evolutionary principles rated most important were antibiotic resistance, environmental mismatch, and somatic selection in cancer, which either occur in a short time frame or are currently visible within society (i.e., obesity) (Hidaka et al., 2015).

However, this trend is not exclusive to the United States. In Germany, the German National Competence‐Based Learning Objectives for Undergraduate Medical Education (NKLM, Nationaler Kom‐petenzbasierter Lernzielkatalog) was passed in 2015 by the Medical Faculty Association (MFT, Medizinischer Fakultätentag) as the official representative body of the medical faculties. This was the first time a nationwide consistent learning objectives catalogue had become available (Fritze et al., 2017). The NKLM includes the Medical Licensing Regulations (ÄAppO, Ärztliche Approbation‐sordnung) previously issued by the government and completely devoid of evolution, in addition to the catalogues of examination‐relevant topics (GK, Gegenstandskataloge) developed by the professional societies, and the Institute for Medical and Pharmaceutical Examination Questions (IMPP, Institutfür Medizinische und Pharmazeutische Prüfungsfragen). Strikingly, the 356 pages NKLM catalogue only includes five references with an evolutionary background (founded in biology and psychiatry).

With the lack of examination questions providing little incentive for premedical students to study evolution, is there any wonder why they leave medical school with no knowledge of evolution.

## 11 POSSIBLE NEXT STEPS

Developing a system of rewards is essential to promote evolution in medicine. As with any new endeavor, the first steps should be attainable, and once achieved, have a clear plan developed to reach its future end goal. The implementation of evolutionary examination questions, particularly for the Medical College Admission Test (MCAT) and the NKLM, is an important first step. This would encourage the addition of evolution to the premedical curriculum and to medical schools. Other possibilities for these early first steps would be to extend the existing networks through joint seminars within the current premedical and medical curriculum, such as biologists and physicians, geologists and nephrologists, and so forth. Publishing companies could also be encouraged to support authors who integrate evolution into textbooks. Developing bibliographies, and publishing and editing on Wikipedia may all prove to be important steps toward creating greater awareness in each subject. It may initially be helpful that in 5 years textbooks will have stickers on their cover denoting “now with evolutionary aspects,” which would hopefully be obsolete in 10 years.

As previously mentioned, the addition of a comprehensive view of the evolution of the healthy human being to the existing networks also serves to bridge the gap between the evolutionary and medical communities. This applies to the EM and evolutionary biology societies, as well as universities, where starting a discussion on a common ground would create an easier transition between the fields. The next steps should be to integrate current subjects from the premedical curriculum with an expanded view of evolution. For instance, the evolution of the organ system and cells would be relevant to anatomy and histology, whereas the evolution of molecules would be relevant to biochemistry.

## 12 FUTURE PERSPECTIVES

Taken together, the importance of a transdisciplinary experience, revealing the perspectives of both fields of thought cannot be underrated. Evolutionary biologists investigate the evolution of the organ system, but usually stop just prior to human emergence, while physicians unknowingly make use of evolution dispersed throughout their investigations of isoenzymes, model organisms, polymorphisms, and so forth, yet are unable to define it. Like a puzzle that cannot be completed until all of its pieces come together, so too must evolution and medicine.

## 13 CONCLUSIONS

Currently, there are fundamental differences that need to be addressed by the evolutionary community (EM and evolution) in order to understand the absence of evolution from medicine. First, the evolutionary fields and medicine have a different perspective of time, which has greatly impacted their respective fields (“why” vs. “how” questions). Secondly, they have a different approach, where evolutionary fields focus on a methodological approach for which its core principles can be broadly applied, while medicine takes a reductionist approach that is content based and specific to humans.

However, while EM applies evolutionary principles, its sole focus on health and disease has caused it to take medicine's reductionist approach. Finally, EM is introduced too late in medical education and should instead begin at the premedical curriculum. Altogether, despite some of the shared challenges between EM and evolution, evolution itself is much easier to integrate into medicine.

To tackle these differences (between evolution and medicine), a commonality is needed through the application of a comprehensive view of the evolution of the healthy human being implemented in the premedical curriculum. However, a common challenge that arises when implementing new subjects into an already existing curriculum is the lack of interdisciplinary discourse. Thus, a system of rewards is needed to motivate actionable change. Ultimately, the goal is for this expanded evolution to be a core science within the medical curriculum and an integral part of medicine. In other words, a redefined evolution in medicine, for which its systematic methods of scientific thinking could produce much more rapid progress for both science and medicine.

## CONFLICT OF INTEREST

The authors declare no competing interests.

## AUTHOR CONTRIBUTIONS

**Jacqueline Moltzau Anderson:** Conceptualization (equal); investigation (equal); project administration (equal); supervision (equal); writing – original draft (equal); writing – review & editing (equal). **Florian Horn:** Conceptualization (equal); investigation (equal); project administration (equal); supervision (equal); writing – original draft (equal); writing – review & editing (equal).

## Data Availability

This paper does not include data.
